# Kidney involvement in myelodysplastic syndromes

**DOI:** 10.1093/ckj/sfae185

**Published:** 2024-06-19

**Authors:** Marie-Camille Lafargue, Jean-Paul Duong Van Huyen, Helmut G Rennke, Marie Essig, Mickaël Bobot, Noémie Jourde-Chiche, Hamza Sakhi, Alexandre KARRAS, Idris Boudhabhay, Philippe Brunet, Hugoline Boulay, Vincent Grobost, Carole Philipponnet, Juliette Jeannel, Jonathan Chemouny, Jean-Jacques Boffa, Dorra Braham-Stambouli, Umut Selamet, Leonardo V Riella, Olivier Fain, Lionel Adès, Pierre Fenaux, Camille Cohen, Arsène Mekinian

**Affiliations:** Department of Nephrology, Tenon's Hospital, Assistance Publique-Hôpitaux de Paris, Université Paris Cité, Paris, France; Department of Pathology, Université Paris Cité, Necker's Hospital, Assistance Publique-Hôpitaux de Paris, Paris, France; Department of Pathology, Brigham and Women's Hospital, Harvard Medical School, Boston, MA, USA; Department of Nephrology, Assistance Publique-Hôpitaux de Paris, Hôpital Ambroise Paré, Boulogne-Billancourt, Université Paris-Saclay, Paris, France; Centre de Néphrologie et Transplantation Rénale, AP-HM, Hôpital de la Conception, CHU de la Conception, Marseille, France; Centre de Néphrologie et Transplantation Rénale, AP-HM, Hôpital de la Conception, CHU de la Conception, Marseille, France; Department of Nephrology, Necker's Hospital, Assistance Publique-Hôpitaux de Paris, Université Paris Cité, Paris, France; Department of Nephrology, CHU HEGP, Paris, France; Department of Nephrology, Necker's Hospital, Assistance Publique-Hôpitaux de Paris, Université Paris Cité, Paris, France; Centre de Néphrologie et Transplantation Rénale, AP-HM, Hôpital de la Conception, CHU de la Conception, Marseille, France; University of Rennes, CHU Rennes, INSERM, EHESP, IRSET (Institut de recherche en santé, environnement et travail), UMR_S 1085, Rennes, France; CHU Estaing, Médecine interne, Clermont-Ferrand, France; Nephrology, Dialysis and Transplantation Department, University Hospital, Clermont-Ferrand, France; Internal Medicine Department, Strasbourg University Hospital, Strasbourg, France; Transplant Unit, Department of Nephrology, University Hospital, Rennes, France; Department of Nephrology, Sorbonne University, Tenon's Hospital, Assistance Publique-Hôpitaux de Paris, Paris, France; Department of Hematology, University Hospital, Rennes, France; Division of Renal Medicine, Brigham and Women's Hospital, Boston, MA, USA; Center for Transplantation Sciences, Division of Nephrology, Massachusetts General Hospital, Harvard Medical School, Boston, MA, USA; Sorbonne Université, Hôpital Saint-Antoine, Service de Médecine Interne et de l'Inflammation-(DHU i2B), Assistance Publique-Hôpitaux de Paris, Paris, France; Hématologie, Hôpital Saint-Louis, Assistance Publique-Hôpitaux de Paris, Université Paris Cité, Paris, France; Hématologie, Hôpital Saint-Louis, Assistance Publique-Hôpitaux de Paris, Université Paris Cité, Paris, France; Service de Néphrologie Hémodialyse, Hôpital Bichat - Claude Bernard, Assistance Publique-Hôpitaux de Paris, Université Paris Cité, Paris, France; Sorbonne Université, Hôpital Saint-Antoine, Service de Médecine Interne et de l'Inflammation-(DHU i2B), Assistance Publique-Hôpitaux de Paris, Paris, France

**Keywords:** acute kidney injury, ANCA-associated vasculitis, auto-immunity, myelodysplastic syndromes, myeloid neoplasms

## Abstract

**Introduction:**

The objective of this study was to describe kidney involvement in patients with myelodysplastic syndromes (MDS), their treatments, and outcomes.

**Methods:**

We conducted a multicenter retrospective study in seven centers, identifying MDS patients with acute kidney injury (AKI), chronic kidney disease (CKD), and urine abnormalities.

**Results:**

Fifteen patients developed a kidney disease 3 months after MDS diagnosis. Median urine protein-to-creatinine ratio was 1.9 g/g, and median serum creatinine was 3.2 mg/dL. Ten patients had AKI at presentation, and 12 had extra-renal symptoms. The renal diagnoses included anti-neutrophil cytoplasmic antibody (ANCA)-associated vasculitis (AAV), ANCA negative vasculitis, C3 glomerulonephritis, immune complex-mediated glomerulonephritis, polyarteritis nodosa, and IgA vasculitis. All patients but one received a specific treatment for the MDS-associated kidney injury. The effect of MDS treatment on kidney injury could be assessed in six patients treated with azacitidine, and renal function evolution was heterogenous. After a median follow-up of 14 months, four patients had CKD stage 3, five had CKD stage 4, and three had end stage kidney disease. On the other hand, three evolved to an acute myeloid leukemia and three died. Compared to 84 MDS controls, patients who had kidney involvement were younger, had a higher number of dysplasia lineages, and were more eligible to receive hypomethylating agents, but no survival difference was seen between the two groups. Compared to 265 AAV without MDS, the ten with MDS-associated pauci-immune vasculitis were older, ANCA serology was more frequently negative, and more cutaneous lesions were seen.

**Conclusion:**

The spectrum of kidney injuries associated with MDS is mostly represented by vasculitis with glomerular involvement, and especially AAV.

KEY LEARNING POINTS **What was known:**

- Hematologic malignancies can be associated with renal complications.
- Renal complications associated with myeloid neoplasms are less described, which is why we studied the clinical and histological presentation of patients with renal impairment during myelodysplastic syndromes.

**This study adds:**

- The kidney, along with other organs, is target of auto-immunity in the setting of myelodysplastic syndromes.
- The spectrum of kidney injuries associated with myelodysplastic syndromes is mostly represented by vasculitis with glomerular involvement, and especially ANCA-associated vasculitis.

**Potential impact:**

- A diagnosis of ANCA-associated vasculitis, in a patient with cytopenias, other than inflammatory anemia, should lead to the research of a possible myelodysplastic syndrome.

## INTRODUCTION

Hematologic malignancies can be associated with renal complications. In the last few years, the concept of monoclonal gammopathy of renal significance (MGRS) has allowed a better characterization of kidney diseases related to monoclonal gammopathies [1]. Nevertheless, renal complications associated with myeloid neoplasms are less described.

Myeloid neoplasms encompass myeloid leukemia, myeloproliferative neoplasms (MPN), myelodysplastic syndromes (MDS), and overlapping syndromes MPN/MDS [2]. MDS are characterized by clonal proliferation of hematopoietic stem cells, recurrent genetic abnormalities, ineffective hematopoiesis, and myelodysplastic features resulting in peripheral cytopenias [3].

MDS can be associated in 10%–25% with systemic inflammatory and auto-immune diseases, among them neutrophilic diseases, connective tissue disorders, arthritis, and vasculitis [4–7]. In two previously retrospective cohorts, the estimated prevalence of glomerulopathies during MDS was between 0.4% and 2.4% [8, 9]. Previous case reports published in the literature mentioned that membranous nephropathy, immunoglobulin-mediated membrano-proliferative glomerulonephritis, and anti-neutrophil cytoplasmic antibody (ANCA)-associated vasculitis (AAV) could be observed among patients with MDS [10–14]. The predominant kidney pathological feature described in a recent series of 19 patients was tubulointerstitial nephritis, which was present in 37% of cases, followed by 16% of ANCA negative vasculitis and 10% of membranous nephropathies [15]. For the remaining patients, the diagnoses were IgA nephropathy, IgA vasculitis, immunoglobulin-associated membrano-proliferative glomerulonephritis type I, crescentic C3 glomerulopathy, fibrillary glomerulonephritis, minimal change disease, and polyarteritis nodosa [15].

The objective of this work was to study the clinical and histological presentation of patients with renal impairment during MDS, as well as their therapeutic management. Furthermore, two analyses were performed: (i) comparison of the hematological phenotype of patients with a kidney injury occurring during MDS with MDS control patients, and (ii) comparison of the renal phenotype of AAV associated with MDS with AAV without MDS.

## MATERIALS AND METHODS

### Patients’ selection

We included patients from three different sources (Supplementary Fig. 1). The first group of patients were recruited at the Brigham and Women's Hospital, and the Massachusetts General Hospital, at Boston, where we screened-in all consecutive patients with a diagnosis of MDS with a kidney biopsy in the last 20 years. The second one included patients with a renal injury related with a diagnosis of vasculitis associated with MDS, from a French multicenter retrospective study by Roupie *et al.* who enrolled patients from different databases conducted by the “Groupe Francophone des Myélodysplasies” (GFM), and “French Network of Dysimmune Disorders Associated with Hemopathies” (MINHEMON) groups [16]. Finally, we reached out to various French scientific societies (“Société Nationale Française de Médecine Interne,” and “Société Francophone de Néphrologie Dialyse et Transplantation”) to do a retrospective collection of patients cases that fitted our inclusion criteria.

Controls with MDS without any kidney injury were extracted from GFM prospective nationwide database. Controls with AAV without any MDS diagnosis come from a French study who included patients with a kidney biopsy analyzed at the Paris Cité Nephropathology unit at Necker hospital [17].

### MDS definitions

Diagnosis criteria for MDS were based on the WHO classification revised in 2016: MDS with single lineage dysplasia (MDS-SLD), MDS with multilineage dysplasia (MDS-MLD), MDS with ring sideroblasts (MDS-RS), MDS-RS with single lineage dysplasia (MDS-RS-SLD), MDS-RS with multilineage dysplasia (MDS-RS-MLD), MDS with isolated del(5q), MDS with excess of blasts (MDS-EB), and unclassifiable MDS (MDS-U) [2]. We excluded patients with MDS secondary to an immunosuppressive treatment or radiotherapy (therapy-related MDS), and MDS secondary to infections, and toxic causes. Cytogenetic analysis and molecular findings were collected when available.

### Definition of kidney diseases

We included adult patients (>18 years old) diagnosed with MDS and a kidney disease. We excluded patients with kidney disease diagnosed before MDS, and remaining stable after the myeloid neoplasm diagnosis.

According to the National Kidney Foundation's Kidney Disease Outcome and Quality Initiative (KDOQI) guidelines, CKD (chronic kidney disease) is defined as either a decreased GFR (glomerular filtration rate <60 ml/min/1.73 m^2^), or markers of kidney damage: albuminuria with an albumin creatinine ratio ≥30 mg/g, urine sediment abnormalities, electrolyte and other abnormalities due to tubular disorders and abnormalities detected by histology among other things [18]. eGFR (estimated glomerular filtration rate) was calculated according to the CKD-EPI formula [19]. Acute kidney injury (AKI) is defined according to the Kidney Disease: Improving Global Outcomes as follows: increase in serum creatinine by ≥0.3 mg/dl (≥26.5 µmol/l) within 48 hours, or increase in serum creatinine to ≥1.5 times baseline, or urine volume <0.5 ml/kg/hour for 6 hours. Kidney biopsies were locally reviewed in each pathology center.

### Clinical data

Clinical and biological data were retrospectively collected from the patients’ charts. Clinical data included were gender, age at the time of the hematological and renal diagnosis, history of hypertension, and diabetes mellitus. Extra-renal organ involvement was recorded if it was directly attributable to systemic manifestations of the MDS.

Biological data, at the time of hematological and renal diagnosis, included blood cell counts, serum creatinine, eGFR, urinary protein-to-creatinine ratio, and urine sediment.

Regarding underlying MDS, data collection included bone marrow cytology and cytogenetic analyses, revised-International Prognosis Scoring System-Revised (IPSS-R) scales and its components, treatments, and the evolution to acute myeloid leukemia (AML). The presence and the type of somatic mutations by next-generation sequencing were recorded when available.

At the time of the renal diagnosis, we collected: antinuclear antibodies and titer, ANCA testing by IF and/or enzyme-linked immunosorbent assay, serum albumin, C-reactive protein (CRP), C3/C4 levels, and the type of treatment.

### Follow-up

During follow-up, events such as dialysis, kidney transplantation, occurrence of AML, or patient death were collected. Evaluation of renal function with proteinuria and serum creatinine was performed at the diagnosis of MDS, at the time of kidney disease, and at last follow-up.

### Statistics

The descriptive data of the patients were expressed in number (frequency) for the binary variables and in median (interquartile range, IQR) for the continuous variables. Owing to non-normal distribution of the values, we used nonparametric tests. Comparisons of continuous variables between two groups were made using the Mann–Whitney test, and those of binary variables were made using the Chi-square test. A Kaplan–Meier method was used to estimate overall survival. The significance level was *P *< .05.

Statistical analyses were performed using GraphPad Prism v.8.00 for MacOS (GraphPad Software, La Jolla, CA, USA).

### Ethics

This study was conducted according to the Declaration of Helsinki and fulfilled the recommendations of the French “Commission Nationale Informatique & Libertés” CNIL. According to its policy, this study was approved by the Institutional Review Board of the Brigham and Women's Hospital (protocol number 2019P003787), who validated a waiver of consent.

## RESULTS

### Characteristics of the patients

Overall, 15 patients [male gender, *n* = 9; median age 76 years 71–80)] with a kidney disease associated with MDS were included in this study.

### Hematological characteristics

The MDS subtypes were MDS-MLD (*n* = 7, 50%), MDS-EB-2 (*n* = 4, 28%), MDS-SLD (*n* = 1, 7%), MDS with isolated del(5q) (*n* = 1, 7%), and MDS-U (*n* = 1, 7%) (Table 1). Median neutrophils count at admission to the renal unit was 2.3 G/l [1.8–3.2]. Median hemoglobin was 9.3 g/dl [7.5–9.8], and median platelets count was 90 G/l [44–219]. Karyotype was available in nine patients, and five had abnormalities including monosomy 7, trisomy 8, and 20q deletion in patient BWH-01; Y chromosome duplication and trisomy 8 in patient FRA-30; del(5q14q35), monosomy 12, add(16), add(17) in patient VAS-03; del(5q) in patient VAS-04; and del(7q) in patient VAS-07. Myeloid somatic mutation analysis was available for two patients: GATA2, DNMT3A, STAG2 (patient MGH-02), WT1 (patient VAS-07). There was no UBA1 mutation in our cohort. Eighty-six percent of the patients had an IPSS-R inferior or equal to 3.5, which was equivalent to very low or low for each of those patients.

**Table 1:** Hematologic and renal characteristics of 15 patients with kidney involvement and MDS.

Pt	Gender Age (y)	MDS type Karyotype Mutations	Hb (g/dl) Plt (G/l) Neutrophils (G/l)	Delay (mo)	SCr (mg/dl)	UPCR (g/g)	Hu	Renal diagnosis	Extra-renal manifestations*
BWH-01	M, 63	MDS-U	7.5	180	3.5	0.7	+	C3 GN	Hemoptysis
		monosomy 7, trisomy 8 and 20q deletion	273						
			3.81						
BWH-02	M, 70	MDS-MLD	6.4	31	1.8	9	+	Immune complex-mediated GN	NA
		Normal	90						
			3						
FRA-07	F, 76	MDS-MLD	9.6	0	4.6	11	+	MPA	Demineralization of both nasal turbinates and septum
		Normal	386						
			8.4						
FRA-10	M, 77	MDS-MLD	10.3	−1	5.5/HD	3	+	MPA	Alveolar hemorrhage
			23						
			1.97						
FRA-15	F, 79	MDS-EB	7.5	1	4.8	0.5	+	ANCA negative vasculitis	NA
			47						
			0.7						
FRA-29	M, 85	MDS-MLD	10.5	0	5.2/HD	NA	+	GPA	Right lower limb purpura
			61						
			NA						
FRA-30	M, 75	MDS-EB	7.4	14	4.6	2.14	+	MPA	NA
		Y chromosome duplication and trisomy 8	40						
			2.7						
FRA-31	F, 59	MDS-EB	9.7	9	0.6	0.15	+	MPA	Alveolar hemorrhage
		Normal	107						
			NA						
FRA-33	F, 78	MDS-SLD	NA	30	3	3	+	MPA	Lower limbs necrotic purpura
MGH-02	M, 28	Mono-MAC syndrome	9.3	0	0.8	3.43	−	ANCA negative vasculitis	Pancreatitis
		GATA2, DNMT3A, STAG2	165						
			1.36						
VAS-01	M, 48	MDS-MLD	NA	47	HD	NA	+	PAN	Subcutaneous knots
VAS-03	M, 73	MDS-EB	NA	0	NA	3	+	C3 GN	NA
		del5q14q35, monosomy 12, add(16), add(17)							
VAS-04	F, 82	MDS-del(5q)	8.3	3	NA	1.14	NA	IgA vasculitis	Purpura, gastric ulcer, and pericarditis
		del5q	334						
			1.9						
VAS-05	M, 69	MDS-MLD	10	7	1.6	0.33	NA	GPA	Purpura, chondritis, scleritis, pericarditis and arthralgia
		Normal	16						
			NA						
VAS-07	F, 85	MDS-MLD	NA	−100	2.1	1.61	NA	MPA	Cough
		del7q							
		WT1							

Abbreviations: F, female; GN, glomerulonephritis; GPA, granulomatosis with polyangiitis; Hb, hemoglobin; HD, hemodialysis; Hu, hematuria; M, male; Mo, months; Mono-MAC syndrome, monocytopenia and mycobacterial infection syndrome; MPA, microscopic polyangiitis; NA, not applicable; PAN, polyarteritis nodosa; Plt, platelets; Pt, patient; SCr, serum creatinine; UPCR, urine protein-to-creatinine ratio; Y, years. *Except altered condition.

### Kidney and systemic presentations

Kidney disease was diagnosed following MDS in eight cases [median time 3 months (0–22)], and concomitantly in six cases. Patients mainly presented with a rapidly progressive glomerulonephritis (*n* = 10, 71.4%), a nephrotic syndrome (*n* = 2, 14.3%), isolated microscopic hematuria in one patient, acute kidney injury associated with proteinuria in one patient, and isolated proteinuria in one patient. Median urine protein-to-creatinine ratio was 1.88 g/g (0.6–3.1) (Table 1). Median serum albumin was 3.2 g/dl (2.7–3.6). Eleven patients presented with a microscopic hematuria. Median serum creatinine was 3.2 mg/dl (1.15–4.56). Ten (66.7%) patients presented with AKI, and three (17.6%) required hemodialysis at diagnosis. Three patients had a previous diagnosis of CKD of unspecified cause with an eGFR between 43 and 51 ml/min/1.73 m^2^.

Auto-immunity was detected in 11 of the 14 tested patients (antinuclear antibodies *n* = 4, and ANCA *n* = 9 with anti-myeloperoxydase (MPO) in five patients, anti-proteinase 3 (PR3) in three patients and no specificity in one case). Out of the six patients with an analysis of the complement, four (66.6%) had a normal serum complement C3, and five (83.3%) a normal serum C4. One patient had a type II cryoglobulinemia. Median CRP was 51 mg/l [15–73.5]. In 12 (80%) cases, extra-renal symptoms were associated; fever and altered condition (*n* = 5, 42%), cutaneous lesions (*n* = 6, 50%), and lung manifestations (*n* = 5, 42%) were the most prevalent.

### Renal diagnoses and histological features of MDS patients

Kidney biopsy was performed in nine (60%) out of the 15 patients. For the other patients, the diagnosis was made according to the clinical and biological data, or based on another organ biopsy, because of kidney biopsy's contraindications (Supplementary Table S1).

**Figure 1:**
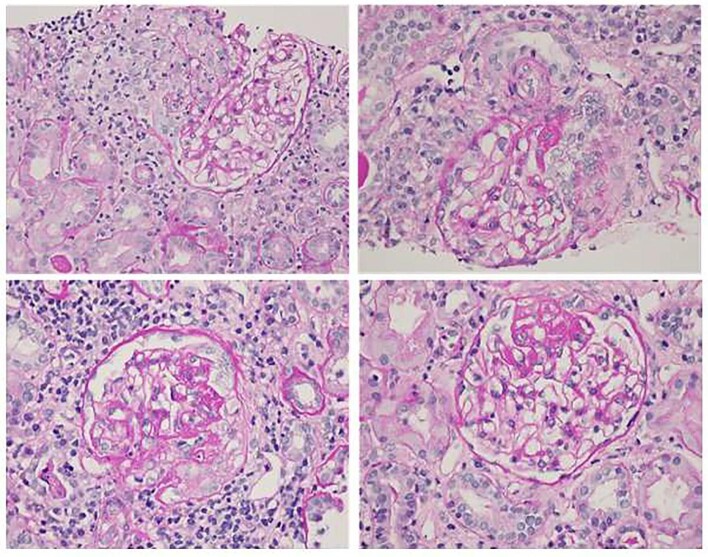
Kidney biopsy of a 28-year-old man with a history of mono-MAC syndrome (monocytopenia and mycobacterial infection syndrome) and MDS referred to renal clinic for evaluation of a nephrotic syndrome with hematuria. Focal proliferative and crescentic glomerulonephritis, pauci-immune cellular crescents and fibrocellular or fibrous crescents with segmental scarring (hematoxylin and eosin staining, magnification ×177). Courtesy of Helmut G Rennke.

For the nine patients who underwent a kidney biopsy, all patients displayed a glomerular involvement, the histological diagnoses were: C3 glomerulonephritis (*n* = 2), immune complex-mediated glomerulonephritis (*n* = 1), microscopic polyangiitis (*n* = 3), pauci-immune necrotizing and crescentic glomerulonephritis (*n* = 1) (Fig. 1), granulomatosis with polyangiitis (*n* = 1), and IgA vasculitis (*n* = 1). The features of light microscopy and immunofluorescence analyses of the kidney biopsies are listed in Table 2.

**Table 2:** Renal pathology description of nine patients with MDS-associated kidney injury.

Patients	Glomeruli	Tubules	Interstitium	Vessels	IF	Diagnosis
BWH-01	37 (9 sclerotic)Rare double contoursInflammatory cells in capillary loopsMesangium hypercellularity	Tubular atrophyEpithelial cell vacuolization	Mild inflammationInfiltrate of mononuclear cells with rare eosinophils and plasma cellsFibrosis 30%	Severe arterial and hyaline arteriolar sclerosis	C3 (3+) in the mesangiumFine granular reactivity for IgA	C3 glomerulonephritis
BWH-02	30 (6 sclerotic).Membrano-proliferativeDiffuse proliferative, and focal crescentic pattern of glomerular injury	Acute tubular injury with focal tubular necrosis	Moderate interstitial inflammation in areas of atrophy, composed of mononuclear cells, few plasma cells, and rare eosinophils	Mild to moderate arterial and arteriolar sclerosis	Granular and smudgy reactivity for IgG (2+), IgA (3+), IgM (1+), C3 (2+), kappa LC (3+) and lambda LC (3+) both along the capillaries and in the mesangium	Immune complex-mediated glomerulonephritis
			Fibrosis 10–15%			
	Active cellular crescents are present in 10 of 24 viable glomeruli					
FRA-07	11 (4 sclerotic)Mesangium hypercellularityFocal segmental extra-capillary proliferation in 1 glomerulusSegmental fibrous lesions with flocculo-capsular synechia in 4 glomeruli	Acute tubular injury with epithelial cell vacuolization	Mild to moderate fibrosisInfiltrate of mononuclear cells	Mild to moderate arterial and arteriolar sclerosis	IgG (3+) along capillary loops. IgG2 (2+), IgG4 (2+), kappa LC (2+) and lambda LC (2+)	Microscopic polyangiitis
FRA-29	18 (3 sclerotic)Cellular crescents in 4 glomeruliFibrous crescents in 3 glomeruliBowman's capsule rupture in 1 glomerulusNo granuloma	Tubular atrophy 10%	Moderate interstitial inflammation, composed of lymphocytes, and few plasma cellsFibrosis 20%	Moderate arterial and arteriolar sclerosis	No significant deposits	Granulomatosis with polyangiitis
FRA-31	14 (1 sclerotic)Endo-capillary proliferationThickening of Bowman's capsule in 1 glomerulus with a segmental lesion associated with a loss of capillary loop lumen	Normal epithelium	No inflammationFibrosis 10%	Mild to moderate arterial and arteriolar sclerosis	Granular and segmental reactivity for IgM (1+) on the Bowman's capsule	Microscopic polyangiitis
MGH-02	Focal proliferative and crescentic glomerulonephritis, pauci-immune cellular crescents are present in 2 of 35 viable glomeruli, and fibrocellular or fibrous crescents are present in 4 glomeruli with segmental scarring	Acute tubular injury with focal tubular necrosis	Chronic-active interstitial nephritis, severe, most likely drug-induced (TMP-SMX)Fibrosis 30%	Mild arterial and arteriolar sclerosis	Fibrin deposits (3+)IgG (2+), kappa LC (2+) and lambda LC (2+) along the capillaries	Pauci-immune necrotizing and crescentic glomerulonephritis
VAS-03	NA	NA	NA	NA	NA	C3 glomerulonephritis
VAS-04	26 (0 sclerotic)Mostly segmental endocapillary proliferation in almost all the glomeruliNo extra-capillary proliferation	NA	Moderate edemaMild fibrosisNo inflammatory infiltrate	Important arterial and arteriolar sclerosis	Subendothelial reactivity for IgA (1+) and IgM (3+)	IgA vasculitis
VAS-07	8 (0 sclerotic)Cellular crescents in 6 glomeruli	Acute tubular injury	Mild fibrosis	Mild arterial and arteriolar sclerosis	No significant deposits	Microscopic polyangiitis

Abbreviations: IF, immunofluorescence; LC, light chain; NA, Detailed report non-available; TMP-SMX, trimethoprim-sulfamethoxazole.

For the patients who did not have a kidney biopsy, two had a diagnosis of microscopic polyangiitis based on the clinical and biological data confirming the ANCA positivity with a MPO specificity, one had an ANCA negative vasculitis with a diagnosis based on an association between AKI, pulmonary involvement, and purpura lesions, one had a granulomatosis with polyangiitis with an ANCA positivity specific to PR3, and one a polyarteritis nodosa based on the imaging (Supplementary Table S1).

Regarding the three patients with a C3 glomerulonephritis, and an immune complex-mediated glomerulonephritis, serum complement C3 and C4 levels were normal. The only patient with a type II cryoglobulinemia had a granulomatosis with polyangiitis.

### Outcomes

At admission, one out of the nine patients with a previous diagnosis of MDS was started on a hematological disease-modifying therapy (azacitidine) 2 months before the renal diagnosis was made. Following the identification of MDS-associated kidney disease, treatment was started or modified in five patients (33.3%), and consisted in azacitidine (*n* = 5), a switch for one patient from lenalidomide to azacitidine, and an allogenic stem cell transplantation (SCT) for another patient (Table 3). Among the six patients with a diagnosis of MDS concomitant or less than two months after renal involvement, three received azacitidine, one underwent an allogenic SCT and one had a withdrawal of azacitidine because of severe infections. After a median follow-up of 14 months (6.6–74.3), three evolved to AML. Hematologic malignancy was considered as stable in seven (46.7%) patients. Three patients died at last follow-up (survival rate 80%).

**Table 3:** Treatments and outcomes of 15 patients with MDS and kidney involvement.

	Hematologic treatments			Renal		Outcomes			
Malignancy	At diagnosis of the kidney disease	After	Follow-up (months)	Diagnosis	Treatment	eGFR at last follow-up	GFR change	Malignancy	Overall
MDS-U	No	No	45.4	C3 GN	Steroids	25	8	AML	Death
MDS-MLD	No	No	2.7	MPGN	Steroids, rituximab, IVIg	30	−7	Stable	Alive
MDS-MLD	No	No	4.9	MPA	Steroids, rituximab	26	14	Stable	Alive
MDS-MLD	No	Azacitidine	3.6	MPA	PLEX, steroids, rituximab	Dialysis	Dialysis	AML	Alive
MDS-EB	Azacitidine	Azacitidine	NA	PiNCG	None^b^	27	16	NA	Alive
MDS-MLD	No	No	0.8	GPA	PLEX, steroids, rituximab	Dialysis	Dialysis	Failure	Death
MDS-EB	No	No	20.2	MPA	Steroids, cyclophosphamide	27	14	Stable	Alive
MDS-EB	No	No	8.4	MPA	Steroids, rituximab	47	-49	Stable	Death
MDS-SLD	No	No	9.3	MPA	Steroids, rituximab	40	26	Stable	Alive
MDS^a^	No	Allogeneic SCT	8	PiNCG	Steroids	83	9	CR	Alive
MDS-MLD	No	Azacitidine	181.9	PAN	Steroids, cyclophosphamide, MMF, rituximab	Dialysis	Dialysis	Stable	Alive
MDS-EB	Azacitidine	No	8.3	C3 GN	Steroids, rituximab, eculizumab	NA	NA	HI	Alive
MDS-del(5q)	Lenalidomide	Azacitidine	14.4	IgA vasculitis	Steroids, MMF	NA	NA	HI	Alive
MDS-MLD	Azacitidine	Azacitidine	45.6	GPA	Steroids, rituximab	44	-29	Stable	Alive
MDS-MLD	Azacitidine	Azacitidine	103.1	MPA	PLEX, steroids, cyclophosphamide	27	15	AML	Alive

Abbreviations: AML, acute myeloid leukemia; CR, complete remission; GN, glomerulonephritis; GPA, granulomatosis with polyangiitis; HI, hematologic improvement; IVIg, intravenous immunoglobulins; MMF, mycophenolate mofetil; MPA, microscopic polyangiitis; MPGN, membrano-proliferative glomerulonephritis; NA, not applicable; PAN, polyarteritis nodosa; PiNCG, pauci-immune necrotizing and crescentic glomerulonephritis; PLEX, plasma exchange; SCT, stem cell transplantation.

a Mono-MAC syndrome.

b None: the patient only received azacitidine for the MDS-associated kidney injury.

All patients but one received a specific treatment for the MDS-associated kidney injury including steroids. The immunosuppressive regimen details are listed in Table 3. Three patients received plasma exchanges, nine had an induction treatment with rituximab, whereas three had cyclophosphamide. After treatment, data on renal function was available for 13 patients. Renal function improved in eight patients (53%), and worsened in three (20%). Among the three patients who underwent renal replacement therapy (hemodialysis) at diagnosis, none recovered any renal function.

### Comparison to AAV without MDS

**Figure 2:**
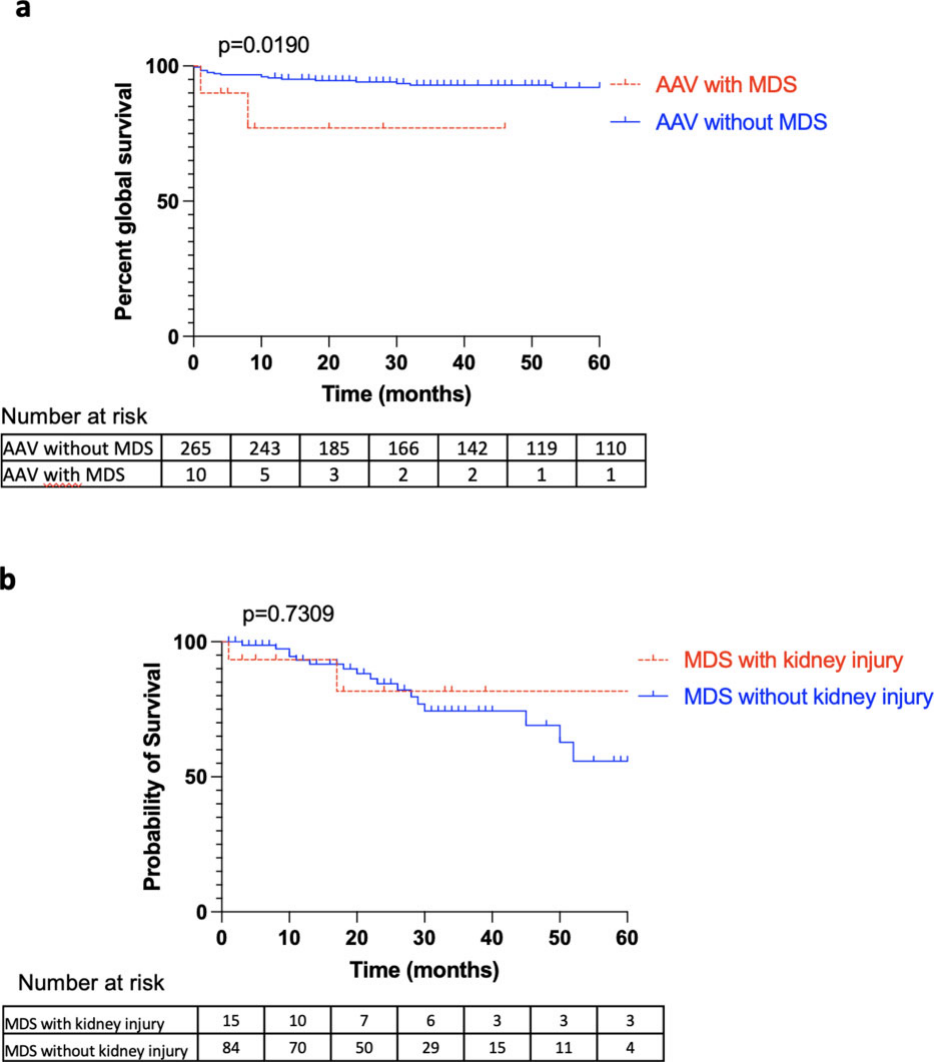
(**a**) Kaplan–Meier curve for the overall survival according to AAV with and without MDS. AAV with MDS group (*n* = 10). AAV without MDS group (*n* = 265). Last follow-up at 5 years. *P *= .0190, log rank test. (**b**) Kaplan–Meier curve for the overall survival according to MDS with and without kidney injury. MDS with kidney injury (*n* = 15). MDS without kidney injury (*n* = 84). Last follow-up at 5 years. *P *= .7309, log rank test.

MDS-associated AAV (*n* = 10) were compared with a cohort of AAV without MDS (*n* = 265) (Table 4). Patients with MDS-associated AAV were older than patients with AAV without MDS (76 vs 63 years old; *P *= .025). The serum creatinine at diagnosis was similar between the two groups (2.5 mg/dl in AAV patients without MDS, and 3.6 mg/dl with MDS; *P *= .781). According to the Berden classification, there was one sclerotic type in the MDS-associated AAV group compared to the AAV without MDS group (*n* = 107, 40.4%). There was no difference in the renal risk score between the two groups (*P *= .523). There is a tendency of a higher frequency of ANCA negative AAV in the MDS-associated AAV group (*n* = 2 vs 11, *P *= .075). Regarding organ involvement, skin was the most prevalent systemic manifestations in patients with MDS-associated AAV, compared with controls AAV (40% vs 9.1%; *P *= .012). In terms of induction regimen, MDS-associated AAV patients were more likely to be treated with rituximab than cyclophosphamide (60% vs 22.3%; *P *= .013 and 30% vs 74.7%; *P *= .005, respectively). It must be noted that the median year at AAV diagnosis was 2018 (2011–2020) for MDS-associated AAV and 2012 (2009–2016) for AAV. The median follow-up for MDS-associated with AAV was 8.5 months (4.8–22), and 46 months (18–98) in AAV controls (*P *< 0.001). Overall survival was significantly decreased in the MDS-associated AAV [median survival not reached in both groups, *P *= .019, odds ratio: 4.43 (95% CI 0.28–76)] (Fig. 2a). Median creatinine at last follow-up was 1.3 mg/dl in the AAV without MDS group, and 1.9 in the AAV with MDS (*P *= .191).

**Table 4:** Characteristics of ANCA-associated vasculitis according to the presence/absence of MDS.

Variable	Total (*n* = 275)	ANCA-associated vasculitis without MDS (*n* = 265)	ANCA-associated vasculitis with MDS (*n* = 10)	*P*
Age (years)	64 [53–74]	63 [53–73]	76 [67–80]	.025
Male	137 (50%)	131 (49.4)	6 (60)	.540
Hypertension	106 (38.8)	102 (38.5)	4 (50)^a^	.715
Diabetes	29 (10.3)	25 (9.4)	3 (37.5)^a^	.0382
ANCA specificity				
Proteinase 3	85 (31)	83 (31.3)	2 (25)	1.000
Myeloperoxydase	177 (64.6)	172 (64.9)	5 (55.6)	.725
None	14 (4.7)	11 (4.2)	2 (20)	.075
CRP (mg/l)	46 [11–127]	46 [10–130]	51 [15–86.5]^a^	.969
Hemoglobin level (g/dl)	9.7 [8.8–11]	9.7 [8.8–11]	9.7 [9.3–10.3]^a^	.836
Serum albumin (g/dl)	3 [2.5–3.4]	2.9 [2.5–3.4]	3.2 [2.7–4]^a^	.24
Renal function				
Serum creatinine (mg/dl)	2.6 [1.4–4.5]	2.5 [1.4–4.5]	3.6 [1.2–4.8]	.781
eGFR (ml/min per 1.73 m^2^)	23 [11–47]	24 [11–46]	14 [12–73]	.846
Dialysis	44 (16)	42 (15.8)	2 (20)	.664
UPCR (g/g)	1.5 [0.9–2.5]	1.5 [0.9–2.5]	2.1 [1.0–3.2]^a^	.278
Positive hematuria	260 (94.5)	254 (95.8)	6 (85.7)^a^	.274
Renal pathology				
Total number of glomeruli	20 [14–25]	20 [14–25]	14 [10–27]^a^	.242
Normal glomeruli <10%	74 (27)	74 (27.9)	0 (0)^a^	.327
Normal glomeruli 10–25%	39 (14.2)	37 (14)	2 (40)^a^	.153
Normal glomeruli >25%	157 (57.1)	154 (58.1)	3 (37.5)^a^	.291
IF/TA ≥25%	107 (38.9)	105 (39.6)	2 (40)^a^	1.000
Berden classification	94 (34.2)	92 (34.7)	2 (40)^a^	.652
Focal	53 (19.3)	52 (19.6)	1 (20)^a^	
Crescentic	64 (23.3)	62 (23.4)	2 (40)^a^	
Mixed	59 (21.5)	59 (22.3)	0 (0)^a^	
Sclerotic	108 (39.3)	107 (40.4)	1 (20)^a^	
ANCA Renal Risk Score	84 (30.5)	81 (30.6)	3 (60)^a^	.523
Low	78 (28.4)	77 (29)	1 (20)^a^	
Moderate				
High				
Organ involvement				
Renal Limited Vasculitis	56 (20.4)	55 (20.8)	1 (10)	.693
Pulmonary involvement	140 (50.9)	136 (51.3)	4 (40)	.535
Cutaneous involvement	28 (10.2)	24 (9.1)	4 (40)	.012
Ophthalmologic involvement	20 (7.3)	19 (7.2)	1 (10)	.536
Ears, nose, and throat	84 (30.5)	82 (30.9)	2 (20)	.728
Cardiac involvement	12 (4.4)	11 (4.2)	1 (10)	.365
Gastrointestinal involvement	9 (3.2)	8 (3.0)	1 (10)	.287
Peripheral nervous system	44 (16)	43 (16.2)	1 (10)	1.000
Central nervous system	6 (2.2)	6 (2.3)	0 (0)	1.000
Induction therapy				
Cyclophosphamide	201 (73.1)	198 (74.7)	3 (30)	.005
Rituximab	65 (23.6)	59 (22.3)	6 (60)	.013
Plasma exchange	50 (18.2)	47 (17.7)	3 (30)	.396
Follow-up (months)	45 [17–94.5]	46 [18–98]	8.5 [4.8–22]	<.001
Relapse during follow-up	57 (20.7)	56 (32.6)	1 (10)	.693
Creatinine at last follow-up (mg/dl)	1.4 [1–2]	1.3 [1–2]	1.9 [1.5–2.3]	.191
Dialysis or kidney transplantation	64 (23.3)	62 (24.7)	2 (20)	1.000
Severe infection	60 (21.8)	58 (23.1)	2 (20)	1.000

Abbreviations: IF/TA, interstitial fibrosis/tubular atrophy; UPCR, urine protein-to-creatinine ratio; Quantitative data are presented as median [interquartile range] or mean (±SD) and qualitative data as *n* (%), as appropriate.

^a^Missing data.

### Comparison between MDS with and without kidney injuries

There were 84 MDS patients without a kidney injury. Compared to these controls, patients who had a kidney involvement were younger (78 vs 75 years, respectively, *P *= .048) (Table 5). Even if there was no difference in prognosis scores (IPSS-R > 3.5 14.3% and 14.3%, *P *= 1), MDS patients with kidney injury had a higher number of dysplastic lineages (two dysplasia, *P *= .044; three dysplasia, *P *= .005). MDS patients with kidney injury were more eligible to receive a specific hematologic treatment with hypomethylating agents (60% and 26.2% in the control MDS group, *P *= .003). The median follow-up from MDS diagnosis was 23 months (12–35) in patients without a kidney injury, and 8 months [3–39] in patients with a kidney injury (*P *= .277). Patients with MDS-associated kidney injury tended to progress more frequently to AML (8.3% and 30%, respectively, *P *= .071). The median survival was 66, and 225 months in MDS with and without kidney injury, respectively [*P *= 0.7309, odd ratio: 0.71 (95% CI 0.24–2.11)] (Fig. 2b).

**Table 5:** Characteristics of MDS according to the presence/absence of a kidney injury.

Variable	Total (*n* = 99)	MDS without kidney injury (*n* = 84)	MDS with kidney injury (*n* = 15)^a^	*P*
Age (years)	78 [74–83]	78 [74–84]	75 [63–79]	.048
Male	61 (61.6%)	52 (61.9)	9 (60)	1.000
MDS type				
MDS-SLD	16 (16.2)	15 (17.9)	1 (7.1)	.455
MDS-MLD	39 (39.4)	32 (38.1)	7 (50)	.803
MDS-SLD-RS	8 (8.1)	8 (9.5)	0 (0)	.597
MDS-MLD-RS	5 (5)	5 (6)	0 (0)	1.000
MDS-EB	18 (18.2)	14 (16.7)	4 (28.6)	.282
MDS-5q-	5 (5)	4 (4.8)	1 (7.1)	.545
MDS-U	8 (8.1)	7 (8.3)	1 (7.1)	1.000
Dysplasia lineage number				
0	54 (58.1)	54 (64.3)	0 (0)	<.001
1	18 (19.4)	16 (19)	2 (22.2)	1.000
2	16 (17.2)	12 (14.3)	4 (44.4)	.044
3	5 (5.4)	2 (2.4)	3 (33.3)	.005
Blasts (%)	3 [1–4]	3 [1–4]	12 [0.5–17.5]	.111
Abnormal karyotype	36 (38.7)	32 (38.1)	4 (44.4)	.731
Neutrophils count (G/L)	2.5 [1.2–4.4]	2.6 [1.4–4.4]	2 [0.68–4.4]	.404
Hemoglobin (g/dL)	10 [9–11.7]	10.1 [9.2–11.8]	9.3 [7.8–10.4]	.044
Platelets (G/L)	139 [98–241]	137 [99–236]	184 [83–317]	.920
IPSS-R	2 [2–3]	2 [2–3]	2.3 [1.8–4]	.985
IPSS-R >3.5	13 (14.3)	12 (14.3)	1 (14.3)	1.000
Specific hematologic treatment	28 (30)	22 (26.2)	6 (60)	.003
Transformation toward AML	10 (10.6)	7 (8.3)	3 (30)	.071
Time between MDS and AML (months)	10 [4.5–20.8]	10 [7–26]	0 [0–19]	.258
Follow-up (months)	23 [11–35]	23 [12–35]	8 [3–39]	.277

Abbreviations: AML, acute myeloid leukemia; IPSS-R, International Prognosis Scoring System-Revised; MDS-5q-, myelodysplastic syndrome with deletion 5q; MDS-EB, MDS with excess of blasts; MDS-MLD, multilineage dysplasia; MDS-RS, MDS with ring sideroblasts; MDS-SLD, single lineage dysplasia; MDS-U, undetermined.

^a^Missing data.

## DISCUSSION

Even if the knowledge about kidney injuries associated with hematological malignancies has increased recently with the concept of MGRS, data about renal complications associated with myeloid neoplasms are scarce, especially in patients with MDS.

In our study, the development of kidney disease tended to be early after MDS diagnosis (3 months), and was concomitant in 40%. This was earlier than in previous large cohort [15]. We identified vasculitis as the most frequent kidney disease associated with MDS, with a high prevalence of AAV. Systemic vasculitis associated with MDS were already described in the literature. However, a recent study reported different patterns of kidney lesions: Schwotzer *et al.* documented the spectrum of kidney diseases from MDS patients with kidney biopsies, and reported that acute tubulointerstitial nephritis was the predominant kidney injury [15]. Yet, tubulointerstitial nephritis is mainly reported in chronic myelomonocytic leukemia, a hematological disorder at the frontier between MDS and myeloproliferative disorders. The fact that we have mostly identified glomerular diseases and systemic vasculitis can be explained by the recruitment of our patients. Indeed, some of our patients were included from a cohort of vasculitis associated with MDS/CMML [16]. Furthermore, in a large cohort studying 123 patients with systemic and auto-immune manifestations associated with MDS/CMML, 32% of the manifestations were systemic vasculitis [6]. Our analysis comparing AAV with and without MDS highlights the fact that we should look for MDS among elderly AAV patients, or with a cutaneous manifestation at AAV diagnosis. In such cases, a bone marrow biopsy should be performed if cytopenia is present.

Even if most of the cases published to date are from small series and case reports, it has also been described that extra-renal vasculitis can be associated with MDS, arguing for the association MDS-crescent glomerulonephritis that we observe here [16]. Whether the mechanisms of lesion development are shared between MDS or classical crescent glomerulonephritis should be explored in a dedicated study.

Hence, auto-immunity seems to be a cornerstone in the setting of kidney injuries related to MDS. Extra-renal manifestations were almost systematically present when the renal disease occurred (80%). Skin (50%) and lungs (42%) were the most prevalent organs involved, with alveolar hemorrhage, purpura, and skin eruption, which was concordant with the Schwotzer *et al.* study in which half of the patients presented extra-renal manifestations [15].

In our study, auto-antibodies were detected in 11 patients, with nine patients having ANCA. Even if antinuclear antibodies were present in 23% of the patients with systemic and auto-immune manifestations related with MDS/CMML in the Mekinian *et al.* study, there was no statistically significant difference in the frequencies of various auto-antibodies in MDS/CMML patients with and without associated auto-immune disorders, except for anti-DNA antibodies [6, 20]. Contrary to CMML patients, MDS patients with auto-immune disorders did not have higher ANCA positivity [20].

In our study, the mortality rate was 20%, with three transformations toward AML, which was similar to another large cohort [15]. Among patients treated with azacitidine, two improved their renal function, and three worsened it, among whom two stayed on long-term hemodialysis. Overall, for the 13 patients with data after the renal treatment, renal function improved in eight patients (53%) and worsened in three (20%). Among the three patients who underwent renal replacement therapy (hemodialysis) at diagnosis, none recovered any renal function. Our results were similar to those of Schwotzer *et al.*, with 10 patients evolving to CKD 3 or 4, and 5 to end stage kidney disease at last follow-up. Thus, which patients with renal complications of MDS would benefit from hypomethylating agents remains to be determined. A prospective phase 2 study sponsored by the GFM assessed azacitidine efficacy and tolerance in patients with MDS and steroids dependent or refractory systemic inflammatory and auto-immune manifestations (SAID) [21]. In this prospective trial, 66% of MDS/CMML patients with SAID obtained SAID complete or partial response after six cycles of azacitidine. Thus, azacitidine may represent an interesting treatment for MDS/CMML patients with associated SAID, acting on both hematological disease and systemic inflammation.

The most represented subtypes of MDS were MDS-MLD and MDS-EB, two subgroups at high risk of transformation toward AML. Nevertheless, our analysis comparing MDS patients with and without kidney injury did not reveal a statistically significant difference in terms of prognosis and overall survival, this may be due to the difference of follow-up between the two groups. However, we noted that patients with a kidney injury tented to be more treated with a hypomethylating agent. Mekinian *et al.* showed in their study enrolling 123 patients, that MDS/CMML associated with systemic inflammatory and auto-immune disorders had worse baseline prognostic factors than control MDS/CMML, but with a similar overall survival [6].

Limitations of this work are mainly due to its retrospective design and the small size of the cohort, even though this is the second largest published study to date. That is why it is not possible to completely rule out the hypothesis of a fortuitous association of renal diseases with MDS. To push the analysis further, the next objectives will be to collect kidney biopsies samples to characterize the pathological and molecular features of the kidney injuries associated with MDS.

The present study clearly indicates that the kidney, along with other organs, is target of auto-immunity in the setting of MDS. MDS are associated with systemic vasculitis, especially with a glomerular involvement such as AAV. A diagnosis of AAV in a patient with cytopenias, other than inflammatory anemia, should lead to research into a possible MDS.

## Supplementary Material

sfae185_Supplemental_File

## Data Availability

The data underlying this article will be shared on reasonable request to the corresponding author.
