# Supplementary material for: Kidney involvement in myelodysplastic syndromes
Source: Clin Kidney J. 2024 Jun 19;17(8):sfae185. doi: 10.1093/ckj/sfae185 (PMC11292217; doi:10.1093/ckj/sfae185)
Supplement: sfae185_Supplemental_File [file sfae185_supplemental_file.docx]

**Supplementary Figure 1:** flowchart. BWH: Brigham and Women’s Hospital. CKD: chronic kidney disease. CMML: chronic myelomonocytic leukemia. GVHD: graft versus host disease. MDS: myelodysplastic syndromes. MGH: Massachusetts General Hospital.

**Supplementary: Table S1: Diagnostic of kidney injury associated to MDS for patients without a kidney biopsy**

| Patient number | Diagnosis | Arguments in favor | Kidney biopsy contraindications |
| --- | --- | --- | --- |
| FRA-10 | Microscopic polyangiitis | Rapidly progressive glomerulonephritis, alveolar hemorrhage, ANCA positivity with myeloperoxydase specificity | Anemia, thrombopenia |
| FRA-15 | ANCA negative vasculitis | AKI, microscopic hematuria, purpura, idiopathic interstitial pneumonia | Obesity |
| FRA-30 | Microscopic polyangiitis | Rapidly progressive glomerulonephritis, ANCA positivity with myeloperoxydase specificity | Thrombopenia |
| FRA-33 | Microscopic polyangiitis | Rapidly progressive glomerulonephritis, ANCA positivity with myeloperoxydase specificity, purpura | Anticoagulant agent for valvular cardiopathy |
| VAS-01 | Polyarteritis nodosa | AKI, kidney’s micro-aneurysms, cutaneous biopsy | Kidney’s micro-aneurysms |
| VAS-05 | Granulomatosis with polyangiitis | Rapidly progressive glomerulonephritis, ANCA positivity with proteinase 3 specificity, chondritis, scleritis, purpura | Thrombopenia |

Abbreviations: AKI: acute kidney injury. ANCA: antineutrophil cytoplasmic antibody. MDS: myelodysplastic syndromes.
